# The impact of early outcome events on the effect of tranexamic acid in post-partum haemorrhage: an exploratory subgroup analysis of the WOMAN trial

**DOI:** 10.1186/s12884-018-1855-5

**Published:** 2018-06-07

**Authors:** Amy Brenner, Haleema Shakur-Still, Rizwana Chaudhri, Bukola Fawole, Sabaratnam Arulkumaran, Ian Roberts, on behalf of the WOMAN Trial Collaborators

**Affiliations:** 1 Clinical Trials Unit, London School of Hygiene and Tropical Medicine, Keppel Street, London, WC1E 7HT UK; 2 Holy Family Hospital, Gynaecology & Obstetrics Unit 1, F-762 Said Pur Road, Satellite Town, Rawalpindi, Pakistan; 3 Department of Obstetrics & Gynaecology, College of Medicine, University of Ibadan, Queen Elizabeth Road, Ibadan, Nigeria; 4 St George’s University of London, Room 1.126, First Floor, Jenner Wing, Cranmer Terrace, London, SW17 0RE UK

**Keywords:** Postpartum haemorrhage, Tranexamic acid, WOMAN trial, Hysterectomy, Death, Bleeding

## Abstract

**Background:**

In severe post-partum haemorrhage, death can occur within hours of bleeding onset so interventions to control the bleeding must be given immediately. In clinical trials of treatments for life-threatening bleeding, established treatments are given priority and the trial treatment is usually given last. However, enrolling patients in whom severe maternal morbidity or death is imminent or inevitable at the time of randomisation may dilute the effects of a trial treatment.

**Methods:**

We conducted an exploratory analysis of data from the WOMAN trial, an international, randomised placebo-controlled trial of the effects of tranexamic acid on death and surgical intervention in 20,060 women with post-partum haemorrhage. We assessed the impact of early maternal death or hysterectomy due to exsanguination on the effect of tranexamic acid on each of these respective outcomes. We conducted repeated analyses excluding patients with these outcomes at increasing intervals from the time of randomisation. We quantified treatment effects using risk ratios (RR) and 99% confidence intervals (CI) and prepared cumulative failure plots.

**Results:**

Among 14,923 women randomised within 3 h of delivery (7518 tranexamic acid and 7405 placebo), there were 216 bleeding deaths (1.5%) and 383 hysterectomies due to bleeding (2.8%). After excluding deaths from exsanguination at increasing time intervals following randomization, there was a significant reduction in the risk of death due to bleeding with tranexamic acid (RR = 0.41; 99% CI 0.19–0.89). However, after excluding hysterectomies at increasing time intervals post-randomization, there was no reduction in the risk of hysterectomy due to bleeding with tranexamic acid (RR = 0.79; 99% CI 0.33–1.86).

**Conclusions:**

Findings from this analysis provide further evidence that tranexamic acid reduces the risk of death from exsanguination in women who experience postpartum haemorrhage. It is uncertain whether tranexamic acid reduces the risk of hysterectomy for bleeding after excluding early hysterectomies.

**Trial registration:**

ISRCTN trial registration number ISRCTN76912190, 8 Dec 2008; ClinicalTrials.gov number NCT00872469, 30 March 2009; PACTR number PACTR201007000192283, 9 Feb 2010; EudraCT number 2008–008441-38, 8 Dec 2010 (retrospectively registered).

**Electronic supplementary material:**

The online version of this article (10.1186/s12884-018-1855-5) contains supplementary material, which is available to authorized users.

## Background

Tranexamic acid reduces bleeding by inhibiting the breakdown of fibrin blood clots. When given prior to incision, tranexamic acid reduces blood loss in elective surgery by about one third [1]. The CRASH-2 trial showed that early tranexamic acid administration reduces death due to bleeding in trauma patients with or at risk of significant haemorrhage [2]. The WOMAN trial assessed the effects of tranexamic acid on death, hysterectomy and other outcomes in 20,060 women with post-partum haemorrhage (PPH). There was a significant reduction in death due to bleeding with tranexamic acid (RR = 0·81, 95% CI 0·65–1·00; *p* = 0·045) [3]. As in traumatic haemorrhage, the reduction was greatest when treatment was given early (within 3 h of delivery), (RR 0·69, 95% CI 0·53–0·90; p = 0·007), with no apparent reduction after 3 h [3, 4]. There was also a decrease in laparotomy to control bleeding in women who received tranexamic acid (RR 0·64, 95% CI 0·49–0·85; p = 0·002). Based on these results, the World Health Organization has recommended the early use (within 3 h of birth) of tranexamic acid for the treatment of PPH [5].

In the WOMAN trial, tranexamic acid did not prevent hysterectomy due to bleeding (RR = 0.95 95%CI 0.78–1.16, *p* = 0.611). During the trial, we noticed that clinicians sometimes decided to perform a hysterectomy at or prior to the time of randomisation and so tranexamic acid could not influence the decision. We predicted that including such hysterectomies as ‘outcome measures’ in the trial would reduce or obscure the effect of tranexamic acid [6].

Inappropriate assumptions about the timing of an exposure’s effect can cause bias towards the null [7]. Even when outcome events occur after randomisation, some will be imminent or inevitable at the time of randomisation and so cannot be prevented by the trial treatment. This is a particular problem in trials in life threatening emergencies when the trial treatment is usually given after the established treatments. Although a trial would ideally evaluate a treatment as it would be used in clinical practice, it is difficult to ensure that a treatment of uncertain effectiveness is given urgently, particularly when clinicians know that half of the patients will receive a placebo.

Given the extent of blood loss in PPH, many of the women enrolled in the WOMAN trial were probably critically ill at the time of randomisation: 59% of women had haemodynamic instability. As such, hysterectomy or death may have been imminent or inevitable in some women. Such outcomes would likely have occurred soon after randomisation. We hypothesised that the inclusion of imminent or inevitable outcome events in the analysis would dilute the treatment effect towards the null. To estimate an undiluted measure of effect, Rothman proposed repeated analyses with varying assumptions about the timing of an exposure’s effect [7]. We aimed to examine whether early outcome events diluted the effect of tranexamic acid on death due to bleeding and hysterectomy due to bleeding by conducting repeated analyses excluding outcomes at increasing intervals from randomisation.

## Methods

The WOMAN trial was a randomised, placebo-controlled trial of the effect of tranexamic acid on death, hysterectomy and other morbidities in women with PPH. It included 20,060 women aged 16 years and older with a clinical diagnosis of PPH recruited from 193 hospitals in 21 countries between 2010 and 2016. We randomly allocated women to receive 1 g of tranexamic acid or placebo by slow intravenous injection. If bleeding continued after 30 min or restarted within 24 h of the first dose, we gave a second dose of 1 g of tranexamic acid or placebo. We obtained follow-up data for 99.8% of patients. We have published full details of the trial rationale, design, methods and results elsewhere [3, 6].

We conducted the trial in accordance with good clinical practice guidelines. The relevant ethics committees and regulatory agencies approved the consent procedures. We obtained informed consent from women if their physical and mental capacity allowed. If a woman could not give consent, we obtained proxy consent from a relative or representative. If no proxy was available, then if local regulation allowed, we deferred or waived the consent. In these cases, we told the woman about the trial as soon as possible and obtained consent for use of the data collected.

### Analysis

We conducted exploratory analyses of the WOMAN trial dataset using the method proposed by Rothman [7]. Our primary outcome was death due to bleeding and our secondary outcome was hysterectomy due to bleeding. We prepared frequency bar charts of the time intervals between randomisation and death due to bleeding and between randomisation and hysterectomy due to bleeding in the treatment and placebo groups to show the time course of bleeding-related outcomes. We then examined the effect of tranexamic acid on these outcomes among women treated within 3 h of delivery since tranexamic acid only appears to be effective when given within this timeframe [3, 4]. We hypothesised that maternal deaths or hysterectomies due to bleeding that occurred soon after randomisation were imminent or inevitable at the time of randomisation. As such, we assessed the impact of early deaths or hysterectomies due to bleeding on the treatment effect by conducting repeated analyses excluding patients with these outcomes at increasing intervals from randomisation. We also excluded patients who died from any cause within the relevant exclusion period, as they could not contribute to the denominator. We increased the length of the exclusion period by one hour at a time, up to 10 h for deaths due to bleeding but 5 h for hysterectomy due to bleeding since there were few hysterectomies beyond 5 h. We excluded hysterectomies completed before randomisation. We conducted intention-to-treat and per-protocol analyses and quantified treatment effects using risk ratios and 99% confidence intervals. We used 99% rather than 95% confidence intervals due to the multiple number of between-group comparisons. We prepared plots of the cumulative percentage of death due to bleeding and hysterectomy due to bleeding in order to supplement the period-specific risk ratios, which can be susceptible to selection bias [8]. We assessed the proportional hazards assumption using the Grambsch-Therneau global test.

To assess the risk of selection bias from post-randomisation exclusions we examined the distribution of baseline characteristics by treatment group. We used stratified analyses to assess potential confounding factors including age, time to treatment, type and place of delivery, cause of haemorrhage, use of uterotonic prophylaxis, estimated blood loss, blood transfusion, and second dose of the trial treatment (or placebo). We adjusted for relevant factors using multivariable log binomial regression and selected a final model using likelihood ratio tests. We also conducted sensitivity analyses of women treated within an hour of delivery, women with uterine atony as the primary cause of haemorrhage, and women who underwent caesarean section.

## Results

In the WOMAN trial, 20,060 women were randomly assigned to receive tranexamic acid (*n* = 10,051) or placebo (*n* = 10,009). After excluding 39 women who did not fulfil the eligibility criteria, withdrew consent or were lost to follow up, data on 20,021 women were available for analysis. Ten women (< 0.1%) had missing data on time of delivery or time of randomisation, so time to treatment was calculated in the remaining 20,011 women. Of these, 14,923 women were randomised within 3 h of delivery (7518 tranexamic acid and 7405 placebo), with a mean time from delivery to randomisation of 1 h (interquartile range = 0.4–1.5 h). Data on time of haemorrhage death were available for all women. Data on time of hysterectomy for bleeding or hysterectomy status were missing for 45 women (0.3%), leaving 14,878 patients for the hysterectomy analyses. Among women randomised within 3 h of delivery, there were 216 deaths due to bleeding (1.5%) and 383 hysterectomies due to bleeding (2.8%). Here we present the results of intention-to-treat analyses. In per-protocol analyses, we excluded 19 women who did not receive tranexamic acid (*n* = 9) or placebo (*n* = 10). The results of the per-protocol analysis were almost identical (see Additional file 1: Tables S1 and S2). The trial arms remained balanced by baseline characteristics (see Additional file 1: Tables S3 and S4), and there was no evidence of confounding (see Additional file 1: Tables S5 and S6).

**Fig. 1:**
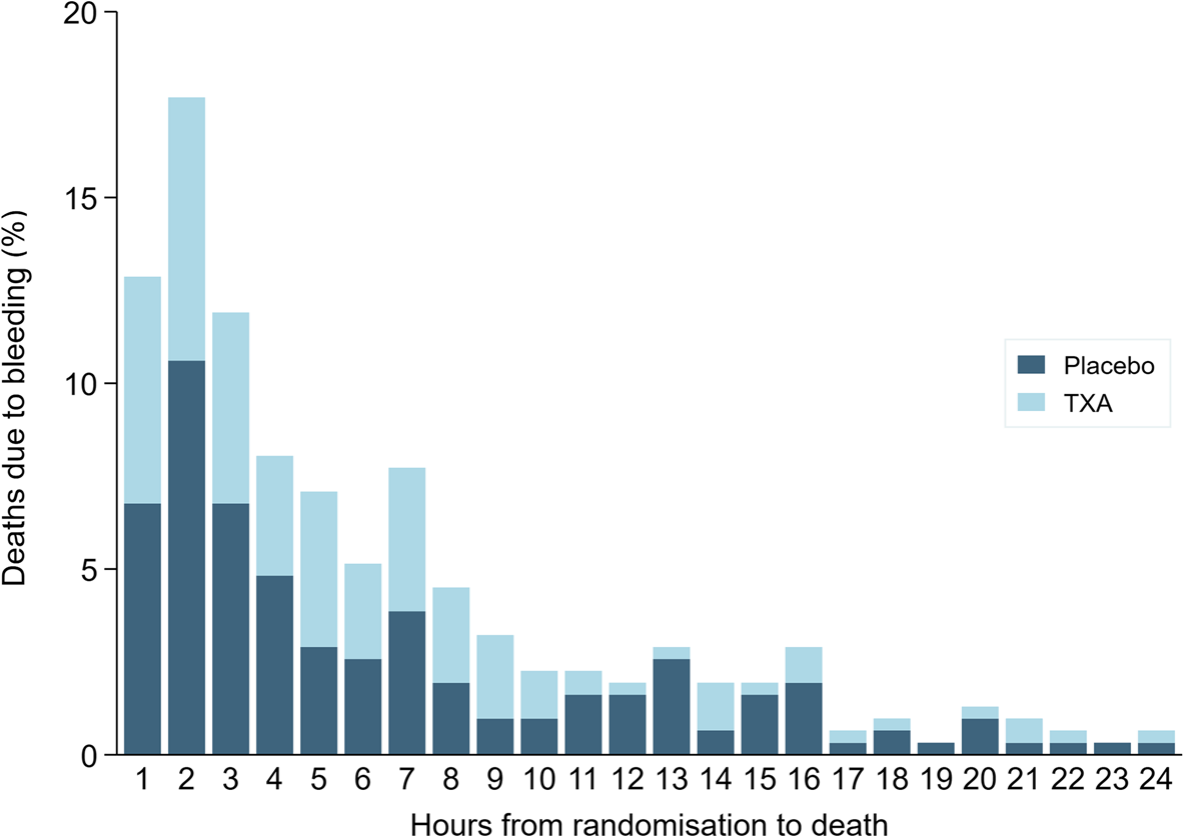
Deaths due to bleeding within 24 h of randomisation by treatment group and hours since randomisation

Figure 1 shows a frequency bar chart of the interval between randomisation and death due to bleeding for the placebo group (*n* = 173) and tranexamic acid group (*n* = 138) over the 24 h after randomisation. The distribution was positively skewed, with 42% of all deaths from exsanguination occurring within 3 h of randomisation, 58% within 5 h, and 80% within 10 h. Thirty-five (10%) deaths from exsanguination occurred more than 24 h after randomisation.

Table 1 shows risk ratios for death due to bleeding in women treated within 3 h of delivery, excluding women who died at increasing intervals from randomisation. When all women were included, there was a 31% reduction in the risk of death due to bleeding with tranexamic acid (RR = 0.69, 99% CI 0.48–0.98). Excluding women who died soon after randomisation increased the treatment effect. The effect was largest after excluding women who died within 9 h of randomisation, with a 59% reduction in death due to bleeding (RR = 0.41, 99% CI 0.19–0.89). Although there was a decreasing trend in risk ratios, the 99% confidence intervals were wide and overlapping. In sensitivity analyses of women treated within an hour of delivery, women with uterine atony and women who underwent caesarean section, we observed the same decreasing trend in risk ratios (see Additional file 1: Tables S7-S9).

**Table 1:** Impact of early deaths due to bleeding on the effect of tranexamic acid

Exclusion interval (hours from randomisation)	Exclusions^a^		N		Death due to bleeding		
	TXA (%)	Placebo (%)	TXA	Placebo	TXA (%)	Placebo (%)	Risk ratio (99% CI)
None	–	–	7518	7405	89 (1.2)	127 (1.7)	0.69 (0.48–0.98)
1	14 (0.2)	15 (0.2)	7504	7390	76 (1.0)	114 (1.5)	0.66 (0.45–0.96)
2	30 (0.4)	38 (0.5)	7488	7367	61 (0.8)	92 (1.3)	0.65 (0.43–1.00)
3	42 (0.6)	57 (0.8)	7476	7348	50 (0.7)	75 (1.0)	0.66 (0.41–1.05)
4	53 (0.7)	70 (1.0)	7465	7335	42 (0.6)	64 (0.9)	0.64 (0.39–1.07)
5	62 (0.8)	77 (1.0)	7456	7328	33 (0.4)	59 (0.8)	0.55 (0.31–0.96)
6	66 (0.9)	85 (1.2)	7452	7320	29 (0.4)	53 (0.7)	0.54 (0.30–0.97)
7	73 (1.0)	94 (1.3)	7445	7311	23 (0.3)	44 (0.6)	0.51 (0.26–0.99)
8	80 (1.1)	97 (1.3)	7438	7308	18 (0.2)	41 (0.6)	0.43 (0.21–0.89)
9	83 (1.1)	101 (1.4)	7435	7304	16 (0.2)	38 (0.5)	0.41 (0.19–0.89)
10	84 (1.1)	104 (1.4)	7434	7301	16 (0.2)	37 (0.5)	0.42 (0.20–0.91)

^a^% is the proportion of the original trial arm excluded (*N* = 7518 TXA, *N* = 7405 placebo). TXA = tranexamic acid. Includes women treated within 3 h of delivery only

**Fig. 2:**
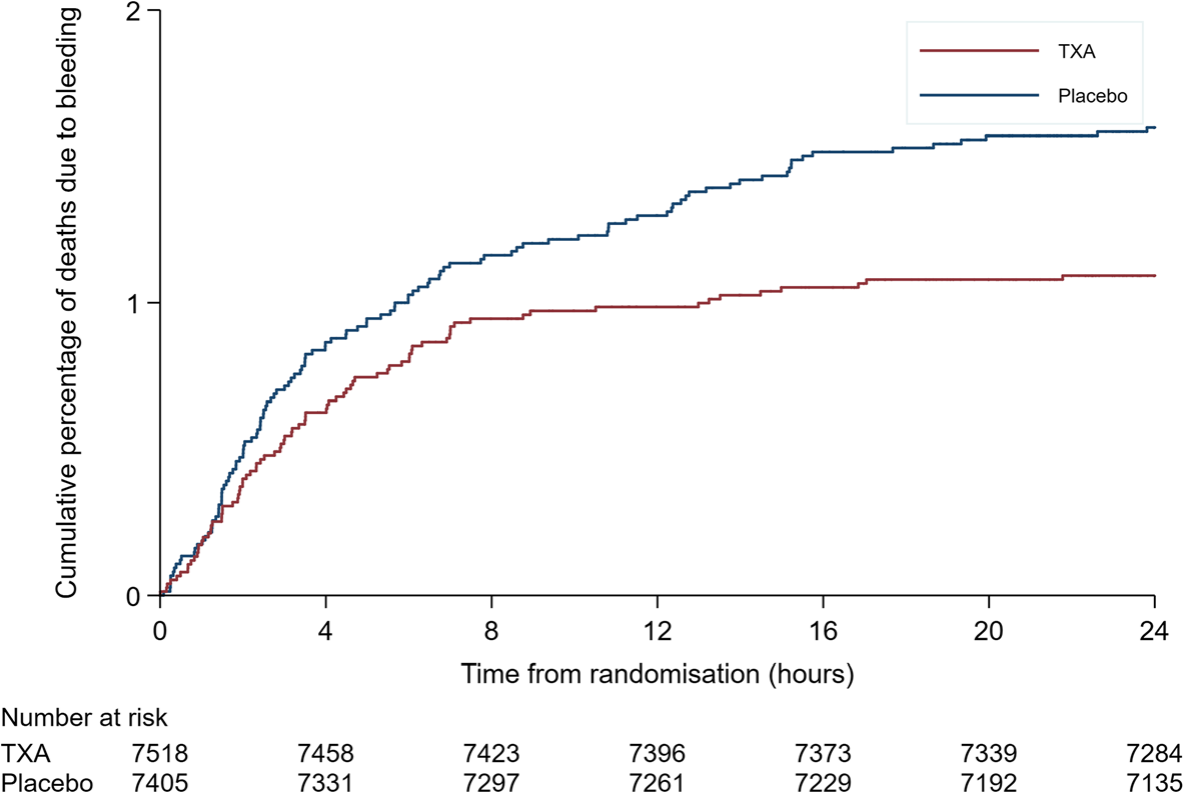
Cumulative percentage of deaths due to bleeding by time from randomisation in the tranexamic acid and placebo group

Figure 2 shows a plot of the cumulative percentage of deaths from bleeding by time from randomisation in the tranexamic acid and placebo groups. For the first few hours after randomisation the curves overlap but later they separate. The Grambsch-Therneau test for proportional hazards gave *p* = 0.06.

**Fig. 3:**
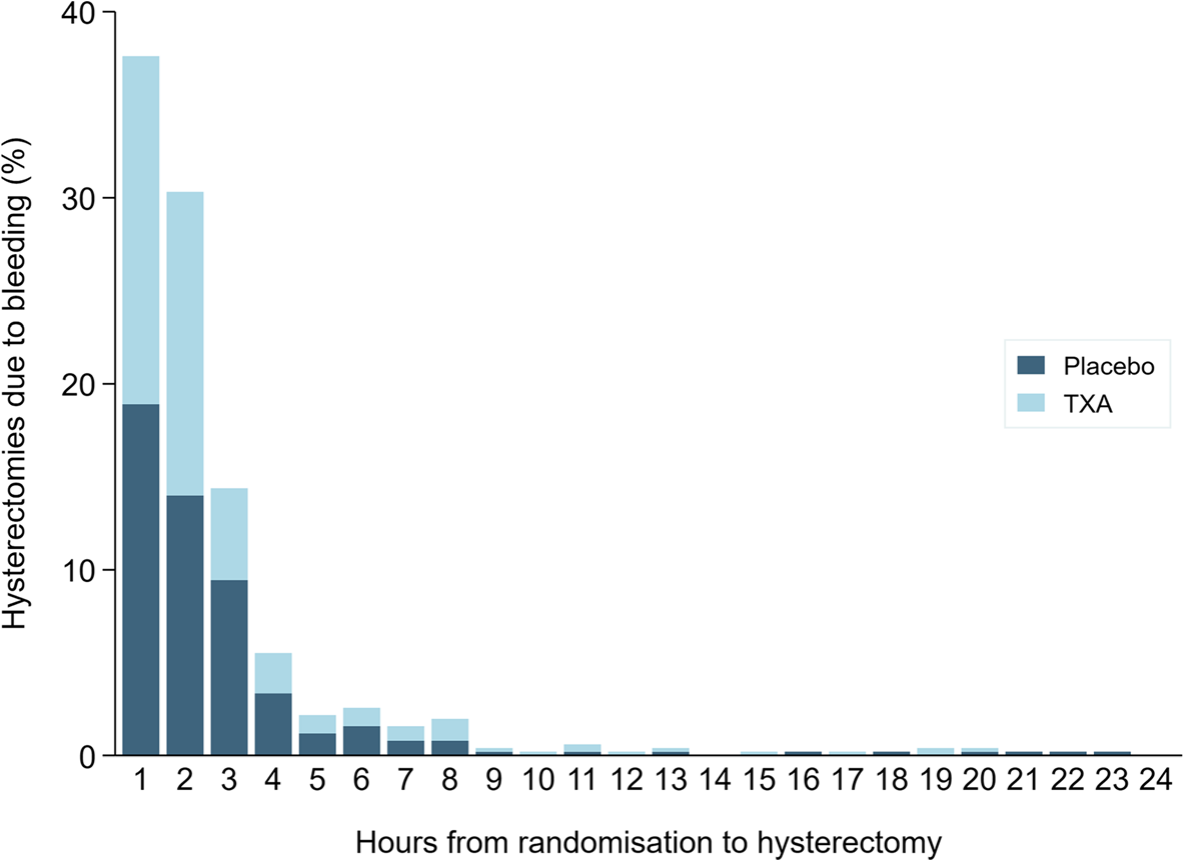
Hysterectomies due to bleeding within 24 h of randomisation by treatment group and hours since randomisation

Figure 3 shows a frequency bar chart of the interval between randomisation and hysterectomy due to bleeding in the placebo group (*n* = 263) and tranexamic acid group (*n* = 245) for the 24 h after randomisation. Again, the distribution was positively skewed with 38% of hysterectomies for bleeding occurring within one hour of randomisation and 82% within 3 h. Less than 2% of hysterectomies for bleeding (*n* = 9) occurred more than 24 h after randomisation.

Table 2 shows risk ratios for hysterectomy due to bleeding for women treated within 3 h of delivery, excluding women who underwent hysterectomy at increasing intervals from randomisation. When all women were included, there was no reduction in the risk of hysterectomy due to bleeding with tranexamic acid (RR = 0.95, 99% CI 0.73–1.23). Excluding women who had a hysterectomy for bleeding soon after randomisation resulted in a decrease in the risk ratio (RR = 0.79; 99% CI 0.33–1.86), however, the 99% confidence intervals overlapped the null at each exclusion interval.

**Table 2:** Impact of early hysterectomies due to bleeding on the effect of tranexamic acid

Exclusion interval (hours from randomisation)	Exclusions^a^		N		Death due to bleeding		
	TXA (%)	Placebo (%)	TXA	Placebo	TXA (%)	Placebo (%)	Risk ratio (99% CI)
None	–	–	7494	7384	188 (2.5)	195 (2.6)	0.95 (0.73–1.23)
1	90 (1.2)	93 (1.3)	7404	7291	112 (1.5)	117 (1.6)	0.94 (0.67–1.32)
2	175 (2.3)	167 (2.3)	7319	7217	42 (0.6)	64 (0.9)	0.65 (0.39–1.08)
3	205 (2.7)	214 (2.9)	7289	7170	23 (0.3)	34 (0.5)	0.67 (0.33–1.33)
4	217 (2.9)	236 (3.2)	7277	7148	19 (0.3)	25 (0.4)	0.75 (0.34–1.63)
5	227 (3.0)	246 (3.3)	7267	7138	16 (0.2)	20 (0.3)	0.79 (0.33–1.86)

^a^% is the proportion of the original trial arm excluded (*N* = 7494 TXA, *N* = 7384 placebo). TXA = tranexamic acid. Includes women treated within 3 h of delivery only

**Fig. 4:**
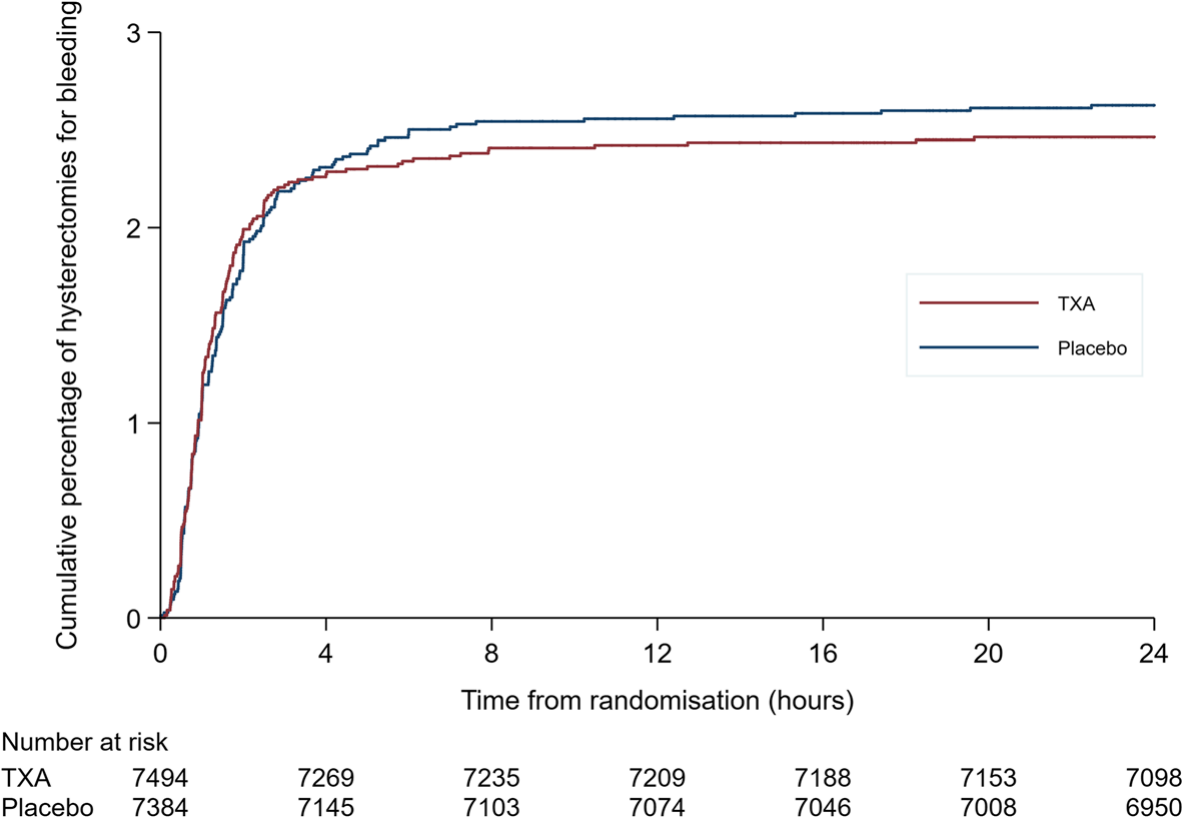
Cumulative percentage of hysterectomies for bleeding by time from randomisation in the tranexamic acid and placebo groups

Figure 4 shows a plot of the cumulative percentage of hysterectomy for bleeding by time from randomisation in the tranexamic acid and placebo groups. In the first hours after randomisation the curves were similar but with minimal separation later. The Grambsch-Therneau test for proportional hazards gave *p* = 0.17.

## Discussion

In the original WOMAN trial, women who experienced PPH were randomized to receive tranexamic acid vs placebo. In the WOMAN trial, we observed a 19% reduction in the risk of death from exsanguination in women who received tranexamic acid compared to placebo, with a 31% reduction in women treated within 3 h of giving birth. In this secondary analysis of WOMAN trial data, after excluding deaths due to bleeding that occurred soon after randomisation, we observed a lower risk of death from exsanguination in women who received early tranexamic acid compared to placebo (RR = 0.41; 99% CI 0.19–0.89). Some women may have been so critically ill at the time of randomisation that death was imminent and inevitable regardless of treatment. The findings of this secondary analysis extend those of the original WOMAN trial by further highlighting the importance of tranexamic acid as an early life-saving intervention for women who experience PPH.

The plasma concentration of tranexamic acid needed to inhibit fibrinolysis is around 5–15 mg/L and tranexamic acid has a half-life of 2–3 h [9–14]. After an intravenous injection of 1 g of tranexamic acid, the plasma concentration should exceed this range for several hours [13, 15]. Because it is eliminated by the kidneys, the concentration could remain elevated for much longer in women with severe bleeding and renal impairment [16]. Further research on the pharmacokinetics and pharmacodynamics of tranexamic acid in obstetric bleeding will help to determine the optimal dosing regimen.

Our analysis has important limitations. Although the statistical analysis plan, which we prepared before seeing the trial results, anticipated that outcomes determined prior to randomisation would dilute the treatment effect, the exploratory analyses presented here were not pre-specified and comprise multiple between-group comparisons. The possibility of a type 1 error cannot be excluded and so our results require cautious interpretation. That said, in keeping with our hypothesis, we observed an increase in the treatment effect on death due to bleeding with an increasing exclusion interval. This finding was consistent in several sensitivity analyses. The temporal distribution of haemorrhage deaths allowed us to exclude women who died soon after randomisation. We did not observe a statistically significant decrease in the risk of hysterectomy for bleeding associated with tranexamic acid compared with placebo after excluding hysterectomies performed early after randomization. Although this finding suggests that tranexamic acid may not decrease the need for hysterectomy as a life-saving surgical intervention for PPH, it is possible that our sample size was inadequate to show a true treatment benefit when excluding early hysterectomies.

Period-specific risk ratios are susceptible to selection bias [8]. Because tranexamic acid reduces deaths due to bleeding, post-randomisation exclusions based on time-to-outcome are not independent of treatment. Indeed, we excluded 20 more deaths from the placebo group than from the treatment group. Although this might be expected to obscure rather than inflate the delayed effects of treatment, because we do not have data on patient characteristics at each time point selection bias remains a concern. Figure 2 provides some unbiased evidence of a lack of treatment benefit early on, in line with our hypothesis that early deaths due to bleeding may dilute the treatment effect, but this may be a spurious finding.

The validity of our results also depend on the accuracy of data on the time of randomisation (treatment) and the time of death but measurement error is inevitable. Although we urged investigators to give the trial treatment as soon as possible after randomisation, some outcomes would have occurred before the treatment was completed. Time of death could have been misclassified since there is often an interval between death and its formal confirmation.

Because maternal death can occur soon after major uncontrolled PPH, interventions to compensate for blood loss (e.g. blood transfusion) and control the bleeding (e.g. hysterectomy) may occur early after PPH diagnosis, often prior to administration of the trial treatment. We conjecture that this may potentially explain the lack of any effect of tranexamic acid on blood transfusion and hysterectomy in the WOMAN trial. However, the results for hysterectomy were inconclusive and we did not have data on time of transfusion. Future studies are needed to examine the effect of tranexamic acid on haemorrhage-related morbidity, and should report the timing of relevant medical and surgical interventions, such as time to first transfusion, uterine balloon tamponade, interventional radiology, and surgical intervention (including hysterectomy and laparotomy).

## Conclusions

In this secondary analysis of data from the WOMAN trial, we observed that tranexamic acid was associated with a reduced risk of maternal death from exsanguination after excluding early maternal deaths from the analysis. This finding is in line with the main findings from the WOMAN trial. Our results suggest that the inclusion of early deaths in the analysis may have diluted the treatment effect of tranexamic acid towards the null. Early outcome events could represent those that are imminent and inevitable. Therefore, the outcomes of some women with life-threatening PPH occurring soon after delivery may not be influenced by exposure to the study drug. These findings also raise the possibility that if we give women tranexamic acid as a first line treatment for PPH rather than a last resort, as now recommended by the World Health Organization [5], its effect on reducing the risk of death due to bleeding may exceed that observed in the WOMAN trial. However, these results should be viewed with caution due to the exploratory nature of this analysis. It remains uncertain whether tranexamic acid reduces the risk of hysterectomy for bleeding after excluding early hysterectomies post-randomisation. Further studies are needed to examine the effect of tranexamic acid on morbidity in PPH.

## Additional file

Additional file 1: Supplementary data analyses. This file provides per protocol analyses (Tables S1 and S2); an assessment of potential selection bias (Tables S3 and S4); an assessment of potential confounding (Tables S5 and S6); a sensitivity analysis of women treated within an hour of delivery (Table S7); a sensitivity analysis of women with uterine atony as the primary cause of haemorrhage (Table S8); a sensitivity analysis of women who underwent caesarean section (Table S9). (DOCX 39 kb)

## Abbreviations

PPH: Post-partum haemorrhage
TXA: Tranexamic acid
WOMAN trial: World Maternal Antifibrinolytic trial
