# Supplementary material for: The impact of early outcome events on the effect of tranexamic acid in post-partum haemorrhage: an exploratory subgroup analysis of the WOMAN trial
Source: BMC Pregnancy Childbirth. 2018 Jun 7;18:215. doi: 10.1186/s12884-018-1855-5 (PMC5992712; doi:10.1186/s12884-018-1855-5)
Supplement: Supplementary file 1 — Supplementary data analyses. This file provides per protocol analyses (Tables S1 and S2); an assessment of potential selection bias (Tables S3 and S4); an assessment of potential confounding (Tables S5 and S6); a sensitivity analysis of women treated within an hour of delivery (Table S7); a sensitivity analysis of women with uterine atony as the primary cause of haemorrhage (Table S8); a sensitivity analysis of women who underwent caesarean section (Table S9). (DOCX 39 kb) [file 12884_2018_1855_MOESM1_ESM.docx]

# **Additional file 1 – Supplementary data analyses**

# **Per protocol analysis**

**Table 1. Impact of early deaths due to bleeding on the effect of tranexamic acid**

| **Exclusion interval**  **(hours from randomisation)** | **Death due to bleeding** | | |
| --- | --- | --- | --- |
|  | **TXA (N=7511)** | **Placebo (N=7396)** | **Risk ratio (99% CI)** |
| **None** | 88 (1.2) | 126 (1.7) | 0.69 (0.48-0.98) |
| **1** | 76 (1.0) | 113 (1.5) | 0.66 (0.45-0.97) |
| **2** | 61 (0.8) | 91 (1.2) | 0.66 (0.43-1.01) |
| **3** | 50 (0.7) | 74 (1.0) | 0.66 (0.42-1.06) |
| **4** | 42 (0.6) | 64 (0.9) | 0.64 (0.39-1.07) |
| **5** | 33 (0.4) | 59 (0.8) | 0.55 (0.31-0.96) |
| **6** | 29 (0.4) | 53 (0.7) | 0.54 (0.30-0.97) |
| **7** | 23 (0.3) | 44 (0.6) | 0.51 (0.26-0.99) |
| **8** | 18 (0.2) | 41 (0.6) | 0.43 (0.21-0.89) |
| **9** | 16 (0.2) | 38 (0.5) | 0.41 (0.19-0.89) |
| **10** | 16 (0.2) | 37 (0.5) | 0.42 (0.20-0.92) |

TXA = tranexamic acid. Includes women treated within 3 hours of delivery only.

**Table 2. Impact of early hysterectomies due to bleeding on the effect of tranexamic acid**

| **Exclusion interval**  **(hours from randomisation)** | **Hysterectomy due to bleeding** | | | |
| --- | --- | --- | --- | --- |
|  | **TXA (N=7489)** | **Placebo (N=7375)** | **Risk ratio (99% CI)** |  |
| **None** | 188 (2.5) | 194 (2.6) | 0.95 (0.74-1.24) |  |
| **1** | 112 (1.5) | 116 (1.6) | 0.95 (0.68-1.33) |  |
| **2** | 42 (0.6) | 63 (0.9) | 0.66 (0.39-1.10) |  |
| **3** | 23 (0.3) | 33 (0.5) | 0.69 (0.34-1.38) |  |
| **4** | 19 (0.3) | 25 (0.4) | 0.75 (0.34-1.63) |  |
| **5** | 16 (0.2) | 20 (0.3) | 0.79 (0.33-1.86) |  |

TXA = tranexamic acid. Includes women treated within 3 hours of delivery only.

# **Assessment of potential selection bias**

**Table 3. Baseline characteristics of women randomised within 3 hours of delivery after exclusion of 163 women who died from bleeding within 10 hours of randomisation**

| **Baseline characteristics** | **Placebo (N = 7301)** | | **TXA (N = 7434)** | | **Total (N = 14735)** |
| --- | --- | --- | --- | --- | --- |
|  | **n** | **(%)** | **n** | **(%)** | **n** |
| **Age (years)** |  |  |  |  |  |
| <16 | 3 | (<0.1) | 1 | (<0.1) | 4 |
| 16-25 | 2559 | (35) | 2554 | (34) | 5113 |
| 26-33 | 3365 | (46) | 3409 | (46) | 6774 |
| >=34 | 1370 | (19) | 1469 | (20) | 2839 |
| Unknown | 4 | (<0.1) | 1 | (<0.1) | 5 |
| **Delivery in hospital** |  |  |  |  |  |
| No | 360 | (5) | 319 | (4) | 679 |
| Yes | 6941 | (95) | 7115 | (96) | 14056 |
| **Type of delivery** |  |  |  |  |  |
| Vaginal | 5050 | (69) | 5055 | (68) | 10105 |
| C-section | 2251 | (31) | 2379 | (32) | 4630 |
| **Time from delivery to randomisation (h)** |  |  |  |  |  |
| <1h | 4673 | (64) | 4799 | (64) | 9472 |
| 1-2h | 1655 | (23) | 1694 | (23) | 3349 |
| 2-3h | 973 | (13) | 941 | (13) | 1914 |
| **Placenta fully delivered** |  |  |  |  |  |
| No | 567 | (8) | 564 | (8) | 1131 |
| Yes | 6734 | (92) | 6870 | (92) | 13604 |
| **Cause of haemorrhage** |  |  |  |  |  |
| Uterine atony | 4718 | (64) | 4797 | (64) | 9515 |
| Placenta praevia or accreta | 745 | (10) | 760 | (10) | 1505 |
| Surgical trauma or tears | 1286 | (18) | 1335 | (18) | 2621 |
| Other | 472 | (6) | 463 | (6) | 935 |
| Unknown | 80 | (1) | 79 | (1) | 159 |
| **Systolic blood pressure (mm Hg)** |  |  |  |  |  |
| >=90 | 6067 | (83) | 6185 | (83) | 12252 |
| <90 | 1230 | (17) | 1248 | (17) | 2478 |
| Unknown | 4 | (<0.1) | 1 | (<0.1) | 5 |
| **Estimated blood loss (ml)** |  |  |  |  |  |
| <=500 | 240 | (3) | 232 | (3) | 472 |
| >500 - 1000 | 3693 | (50) | 3751 | (50) | 7444 |
| >1000 - 1500 | 2087 | (29) | 2094 | (28) | 4181 |
| >1500 | 1281 | (18) | 1356 | (18) | 2637 |
| Unknown | 0 | (0) | 1 | (<0.1) | 1 |
| **Uterotonic prophylaxis** |  |  |  |  |  |
| No | 59 | (1) | 46 | (1) | 105 |
| Yes | 7156 | (98) | 7311 | (98) | 14467 |
| Unknown | 86 | (1) | 77 | (1) | 163 |
| **Clinical signs of haemodynamic instability** |  |  |  |  |  |
| No | 3272 | (45) | 3292 | (44) | 6564 |
| Yes | 4029 | (55) | 4142 | (56) | 8171 |
| **Second dose received** |  |  |  |  |  |
| No | 5151 | (70) | 5286 | (71) | 10437 |
| Yes | 2150 | (29) | 2144 | (29) | 4294 |
| Unknown | 0 | (0) | 4 | (<0.1) | 4 |

TXA = tranexamic acid

**Table 4. Baseline characteristics of women randomised within 3 hours of delivery after the exclusion of 347 women who underwent a hysterectomy for bleeding within 10 hours of randomisation**

| **Baseline characteristics** | **Placebo (N=7138)** | | **TXA (N=7267)** | | **Total (N=14405)** |
| --- | --- | --- | --- | --- | --- |
|  | **n** | **(%)** | **n** | **(%)** | **n** |
| **Age (years)** |  |  |  |  |  |
| <16 | 3 | (0) | 1 | (<0.1) | 4 |
| 16-25 | 2538 | (36) | 2533 | (35) | 5071 |
| 26-33 | 3288 | (46) | 3332 | (46) | 6620 |
| >=34 | 1305 | (18) | 1400 | (19) | 2705 |
| Unknown | 4 | (<0.1) | 1 | (<0.1) | 5 |
| **Delivery in hospital** |  |  |  |  |  |
| No | 355 | (5) | 312 | (4) | 667 |
| Yes | 6783 | (95) | 6955 | (96) | 13738 |
| **Type of delivery** |  |  |  |  |  |
| Vaginal | 4990 | (70) | 4995 | (69) | 9985 |
| C-section | 2148 | (30) | 2272 | (31) | 4420 |
| **Time from delivery to randomisation (h)** |  |  |  |  |  |
| <1h | 4561 | (64) | 4676 | (64) | 9237 |
| 1-2h | 1624 | (23) | 1667 | (23) | 3291 |
| 2-3h | 953 | (13) | 924 | (13) | 1877 |
| **Placenta fully delivered** |  |  |  |  |  |
| No | 540 | (8) | 538 | (7) | 1078 |
| Yes | 6598 | (92) | 6729 | (93) | 13327 |
| **Cause of haemorrhage** |  |  |  |  |  |
| Uterine atony | 4631 | (65) | 4719 | (65) | 9350 |
| Placenta praevia or accreta | 691 | (10) | 699 | (10) | 1390 |
| Surgical trauma or tears | 1271 | (18) | 1320 | (18) | 2591 |
| Other | 468 | (7) | 451 | (6) | 919 |
| Unknown | 77 | (1) | 78 | (1) | 155 |
| **Systolic blood pressure (mm Hg)** |  |  |  |  |  |
| >=90 | 5958 | (83) | 6085 | (8) | 12043 |
| <90 | 1176 | (16) | 1181 | (16) | 2357 |
| Unknown | 4 | (<0.1) | 1 | (<0.1) | 5 |
| **Estimated blood loss (ml)** |  |  |  |  |  |
| <=500 | 240 | (3) | 232 | (3) | 472 |
| >500 - 1000 | 3674 | (51) | 3729 | (51) | 7403 |
| >1000 - 1500 | 2051 | (29) | 2049 | (28) | 4100 |
| >1500 | 1173 | (16) | 1256 | (17) | 2429 |
| Unknown | 0 | (0) | 1 | (<0.1) | 1 |
| **Uterotonic prophylaxis** |  |  |  |  |  |
| No | 59 | (1) | 42 | (1) | 101 |
| Yes | 6995 | (98) | 7150 | (98) | 14145 |
| Unknown | 84 | (1) | 75 | (1) | 159 |
| **Clinical signs of haemodynamic instability** |  |  |  |  |  |
| No | 3241 | (45) | 3269 | (45) | 6510 |
| Yes | 3897 | (55) | 3998 | (55) | 7895 |
| **Second dose received** |  |  |  |  |  |
| No | 5122 | (72) | 5251 | (72) | 10373 |
| Yes | 2016 | (28) | 2016 | (28) | 4032 |

TXA = tranexamic acid

# **Assessment of potential confounding**

**Table 5. Impact of early deaths due to bleeding on the effect of tranexamic acid before and after adjustment for potential confounders**

| **Exclusion interval (hours from randomisation)** | **Death due to bleeding** | |
| --- | --- | --- |
|  | **Crude risk ratio (99% CI)** | **Adjusted risk ratio* (99% CI)** |
|  |  |  |
| **None** | 0.69 (0.48-0.98) | 0.72 (0.51-1.02) |
| **1** | 0.66 (0.45-0.96) | 0.68 (0.47-0.99) |
| **2** | 0.65 (0.43-1.00) | 0.67 (0.44-1.02) |
| **3** | 0.66 (0.41-1.05) | 0.67 (0.42-1.07) |
| **4** | 0.64 (0.39-1.07) | 0.66 (0.40-1.09) |
| **5** | 0.55 (0.31-0.96) | 0.56 (0.32-0.97) |
| **6** | 0.54 (0.30-0.97) | 0.54 (0.30-0.98) |
| **7** | 0.51 (0.26-0.99) | 0.52 (0.27-1.01) |
| **8** | 0.43 (0.21-0.89) | 0.44 (0.21-0.91) |
| **9** | 0.41 (0.19-0.89) | 0.42 (0.20-0.91) |
| **10** | 0.42 (0.20-0.91) | 0.43 (0.20-0.93) |

*Adjusted for age, cause of haemorrhage, transfusion, second dose and type of delivery. Includes women treated within 3 hours of delivery only.

**Table 6. Impact of early hysterectomies due to bleeding on the effect of tranexamic acid before and after adjustment for potential confounders**

| **Exclusion interval (hours from randomisation)** | **Hysterectomy due to bleeding** | |
| --- | --- | --- |
|  | **Crude risk ratio (99% CI)** | **Adjusted risk ratio* (99% CI)** |
|  |  |  |
| **None** | 0.95 (0.73-1.23) | 0.96 (0.75-1.23) |
| **1** | 0.94 (0.67-1.32) | 0.97 (0.70-1.35) |
| **2** | 0.65 (0.39-1.08) | 0.67 (0.41-1.11) |
| **3** | 0.67 (0.33-1.33) | 0.68 (0.34-1.36) |
| **4** | 0.75 (0.34-1.63) | 0.77 (0.35-1.67) |
| **5** | 0.79 (0.33-1.86) | 0.80 (0.34-1.89) |

*Adjusted for age, second dose and type of delivery. Includes women treated within 3 hours of delivery only.

# **Sensitivity analysis – women treated within an hour of delivery**

**Table 7 Impact of early deaths due to bleeding on the effect of tranexamic acid among women treated within an hour of delivery**

| **Exclusion interval (hours from randomisation)** | **Exclusions*** | | **N** | | **Death due to bleeding** | | |
| --- | --- | --- | --- | --- | --- | --- | --- |
|  | **TXA (%)** | **Placebo (%)** | **TXA** | **Placebo** | **TXA (%)** | **Placebo (%)** | **Risk ratio (99% CI)** |
| **None** | - | - | 4844 | 4723 | 49 (1.0) | 60 (1.3) | 0.80 (0.49-1.30) |
| **1** | 9 (0.2) | 5 (0.1) | 4835 | 4718 | 41 (0.9) | 56 (1.2) | 0.71 (0.42-1.21) |
| **2** | 18 (0.4) | 18 (0.4) | 4826 | 4705 | 32 (0.7) | 43 (0.9) | 0.73 (0.40-1.32) |
| **3** | 23 (0.5) | 27 (0.6) | 4832 | 4699 | 28 (0.6) | 36 (0.8) | 0.76 (0.40-1.45) |
| **4** | 30 (0.6) | 37 (0.8) | 4814 | 4686 | 24 (0.5) | 28 (0.6) | 0.83 (0.41-1.70) |
| **5** | 36 (0.7) | 40 (0.9) | 4808 | 4683 | 18 (0.4) | 26 (0.6) | 0.67 (0.31-1.48) |
| **6** | 37 (0.8) | 43 (0.9) | 4807 | 4680 | 17 (0.4) | 24 (0.5) | 0.69 (0.31-1.56) |
| **7** | 42 (0.9) | 48 (1.0) | 4802 | 4675 | 12 (0.3) | 19 (0.4) | 0.62 (0.24-1.59) |
| **8** | 44 (0.9) | 48 (1.0) | 4800 | 4675 | 11 (0.2) | 19 (0.4) | 0.56 (0.21-1.49) |
| **9** | 45 (0.9) | 49 (1.0) | 4799 | 4674 | 10 (0.2) | 18 (0.4) | 0.54 (0.20-1.49) |
| **10** | 45 (0.9) | 50 (1.1) | 4799 | 4673 | 10 (0.2) | 18 (0.4) | 0.54 (0.20-1.49) |

*% is the proportion of the original trial arm excluded (N=4844 TXA, N=4723 placebo). TXA = tranexamic acid.

# **Sensitivity analysis – women with uterine atony as the primary cause of haemorrhage**

**Table 8 Impact of early deaths due to bleeding on the effect of tranexamic acid among women with uterine atony as the primary cause of haemorrhage**

| **Exclusion interval (hours from randomisation)** | **Exclusions*** | | **N** | | **Death due to bleeding** | | |
| --- | --- | --- | --- | --- | --- | --- | --- |
|  | **TXA (%)** | **Placebo (%)** | **TXA** | **Placebo** | **TXA (%)** | **Placebo (%)** | **Risk ratio (99% CI)** |
| **None** | - | - | 4840 | 4774 | 46 (1.0) | 71 (1.5) | 0.64 (0.39-1.04) |
| **1** | 8 (0.2) | 6 (0.1) | 4832 | 4768 | 38 (0.8) | 66 (1.4) | 0.57 (0.34-0.96) |
| **2** | 16 (0.3) | 20 (0.4) | 4824 | 4754 | 30 (0.6) | 53 (1.1) | 0.56 (0.31-1.00) |
| **3** | 20 (0.4) | 33 (0.7) | 4820 | 4741 | 26 (0.5) | 42 (0.9) | 0.61 (0.32-1.16) |
| **4** | 26 (0.6) | 39 (0.8) | 4813 | 4735 | 20 (0.4) | 36 (0.8) | 0.55 (0.27-1.12) |
| **5** | 32 (0.7) | 43 (0.9) | 4808 | 4731 | 15 (0.3) | 33 (0.7) | 0.45 (0.20-1.00) |
| **6** | 33 (0.7) | 45 (0.9) | 4807 | 4729 | 14 (0.3) | 31 (0.7) | 0.44 (0.19-1.02) |
| **7** | 37 (0.8) | 49 (1.0) | 4803 | 4725 | 10 (0.2) | 27 (0.6) | 0.36 (0.14-0.94) |
| **8** | 42 (0.9) | 51 (1.1) | 4798 | 4723 | 7 (0.2) | 25 (0.5) | 0.28 (0.09-0.83) |
| **9** | 43 (0.9) | 54 (1.1) | 4797 | 4720 | 7 (0.2) | 23 (0.5) | 0.30 (0.10-0.91) |
| **10** | 43 (0.9) | 56 (1.2) | 4797 | 4718 | 7 (0.2) | 22 (0.5) | 0.31 (0.10-0.96) |

*% is the proportion of the original trial arm excluded (N=4840 TXA, N=4774 placebo). TXA = tranexamic acid. Includes women treated within 3 hours of delivery only.

# **Sensitivity analysis – women who underwent caesarean section**

**Table 9 Impact of early deaths due to bleeding on the effect of tranexamic acid among women who underwent caesarean section**

| **Exclusion interval (hours from randomisation)** | **Exclusions*** | | **N** | | **Death due to bleeding** | | |
| --- | --- | --- | --- | --- | --- | --- | --- |
|  | **TXA (%)** | **Placebo (%)** | **TXA** | **Placebo** | **TXA (%)** | **Placebo (%)** | **Risk ratio (99% CI)** |
| **None** | - | - | 2404 | 2277 | 23 (1.0) | 36 (1.6) | 0.61 (0.31-1.20) |
| **1** | 2 (0.1) | 3 (0.1) | 2402 | 2274 | 21 (0.9) | 33 (1.5) | 0.60 (0.29-1.23) |
| **2** | 5 (0.2) | 9 (0.4) | 2399 | 2268 | 18 (0.8) | 28 (1.2) | 0.61 (0.28-1.31) |
| **3** | 9 (0.4) | 11 (0.4) | 2395 | 2266 | 14 (0.6) | 26 (1.2) | 0.51 (0.22-1.19) |
| **4** | 11 (0.5) | 13 (0.6) | 2393 | 2264 | 13 (0.5) | 24 (1.1) | 0.51 (0.21-1.24) |
| **5** | 14 (0.6) | 16 (0.7) | 2390 | 2261 | 10 (0.4) | 21 (0.9) | 0.45 (0.17-1.21) |
| **6** | 15 (0.6) | 17 (0.8) | 2389 | 2260 | 9 (0.4) | 20 (0.9) | 0.43 (0.15-1.19) |
| **7** | 20 (0.8) | 21 (0.9) | 2384 | 2256 | 5 (0.2) | 16 (0.7) | 0.30 (0.08-1.10) |
| **8** | 23 (1.0) | 23 (1.0) | 2381 | 2254 | 3 (0.1) | 14 (0.6) | 0.20 (0.04-1.04) |
| **9** | 25 (1.0) | 24 (1.1) | 2379 | 2253 | 1 (<0.1) | 13 (0.6) | 0.07 (0.01-1.05) |
| **10** | 25 (1.0) | 26 (1.1) | 2379 | 2251 | 1 (<0.1) | 13 (0.6) | 0.07 (0.01-1.05) |

*% is the proportion of the original trial arm excluded (N=2404 TXA, N=2277 placebo). TXA = tranexamic acid. Includes women treated within 3 hours of delivery only.
